# THE IMPACT OF SOCIAL DETERMINANTS OF HEALTH ON DETECTING DEMENTIA: REFRAMING HEALTH EQUITY

**DOI:** 10.1093/geroni/igad104.0273

**Published:** 2023-12-21

**Authors:** Joshua Chodosh, Soo Borson

**Affiliations:** NYU Grossman School of Medicine, New York City, New York, United States; University of Southern California, Los Angeles, California, United States

## Abstract

Social determinants of health (SDoH) contribute to the incidence of Alzheimer’s disease and related dementia (ADRD) and to missed opportunities to reduce the impact of its symptoms. They also play a fundamental role in detection of ADRD, leading to disparities in diagnosis as well as care. Public health practitioners have an outsize role in addressing the intersection between ADRD prevalence and related SDoH, especially for persons from minoritized and under-resourced communities. We will delineate the effects of this intersectionality and the need to reframe dementia detection strategies to promote health equity, reduce related stigma, and repair the mistrust that is integral to care inequities. We will highlight the ongoing efforts of our public health partners who are committed to ensuring that dementia is detected before a crisis occurs and opportunities they offer to support the efforts of our PHCOE partners in dementia prevention and caregiving.

